# The value of LAM and LprG in extracellular vesicles in the diagnostic and therapeutic field of renal tuberculosis

**DOI:** 10.3389/ftubr.2025.1608780

**Published:** 2025-08-12

**Authors:** Xuefeng Peng, Yue Li, Suiyang Jin, Qiang Wang

**Affiliations:** ^1^Guizhou Medical University, Guiyang, Guizhou, China; ^2^Department of Urology, Guizhou Medical University Affiliated Hospital, Guiyang, Guizhou, China

**Keywords:** extracellular vesicles, Mycobacterium tuberculosis, renal tuberculosis, LAM, LprG, diagnosis and treatment

## Abstract

Extracellular vesicles have been a hot research topic in recent years, and the diagnostic and therapeutic value of LprG and LAM, two key markers present in extracellular vesicles secreted by Mycobacterium tuberculosis or Mycobacterium tuberculosis-infected immune cells, in tuberculosis, has been widely emphasized in recent years. Genitourinary tuberculosis is a common form of extrapulmonary tuberculosis, and renal tuberculosis accounts for more than 20% of patients with Genitourinary tuberculosis. In this paper, we summarize the findings and research ideas of LprG and LAM in the diagnosis and treatment of renal tuberculosis in recent years and conclude that LprG and LAM have their unique diagnostic value in the intrapulmonary and extrapulmonary fields and can be used as a new potential idea for vaccine or immunotherapy in the future for research.

## 1 Research background and significance

In 2022, tuberculosis (TB) remained the second leading cause of death from a single infectious pathogen following COVID-19. According to the World Health Organization's statistics for 2022, ~1.3 million people worldwide died from tuberculosis, with China accounting for more than 7% of the global case burden (1). The high transmissibility and the prolonged treatment duration of tuberculosis result in higher costs for patients, leading to significant financial burdens for many families. Renal tuberculosis, as one of the common forms of extra pulmonary tuberculosis, is often overlooked by patients due to the difficulty in early diagnosis and atypical clinical manifestations. This results in renal tuberculosis patients seeking medical care at a later stage, making treatment more challenging and leading to poorer patient outcomes (2). With the increasing number of renal tuberculosis cases among children, the elderly, pregnant women, and individuals infected with HIV, the development of a strategy for early diagnosis and screening, as well as precise treatment for renal tuberculosis, has become increasingly important.

Renal tuberculosis is primarily a secondary condition resulting from the hematogenous dissemination of Mycobacterium tuberculosis from pulmonary tuberculosis lesions, with only a small proportion of cases being primary renal tuberculosis due to compromised immunity for various reasons. Urine acid-fast bacilli smear microscopy is a commonly used clinical method for detecting renal tuberculosis, similar to the World Health Organization (WHO)- recommended GeneXpert MTB/RIF test. Both methods have relatively poor sensitivity, with the former achieving a sensitivity of only 40% and the latter a positive detection rate of around 60% at best. Histopathological biopsy and serum or urine Mycobacterium tuberculosis culture remains the gold standard for diagnosing renal tuberculosis. However, the former is invasive, and the latter requires a long waiting time and is challenging to collect samples, making them not the preferred method for clinical diagnosis (3). With the advancement of imaging technology, a series of techniques, including cystoscopy, urethrography, B-ultrasound, CT scan, and MRI scan, have improved the detection rate of renal tuberculosis. However, imaging examinations cannot serve as the gold standard for diagnosing renal tuberculosis but rather as an indirect diagnostic method or for exclusionary purposes (4).

In recent years, technologies such as quantitative polymerase chain reaction (qPCR) gene detection have been used to analyze and assess the occurrence, development, and progression of tuberculosis (5). However, due to the complexity of tuberculosis in primary clinical settings, the diagnostic efficacy remains unsatisfactory. Therefore, the search for more specific DNA, RNA, or protein biomarkers of Mycobacterium tuberculosis (Mtb) has become a hot topic in recent research, and extracellular vesicles have naturally come into focus (6).

Extracellular Vesicles (EVs) are a class of tiny vesicles released by living cells, enclosed by a lipid bilayer that contains their contents and is unable to self-replicate. Depending on their size, they can be classified as ectosomes or exosomes. The contents they carry vary depending on the cell's life cycle and biological state (7), including proteins, nucleic acids, lipids, sugars, and other substances. They act independently or synergistically to mediate various biological functions in the body, such as cell-to-cell signaling, cell cycle regulation and differentiation, immune modulation, tissue repair, and cellular waste elimination (8).

In recent studies, it has been found that human infection with Mycobacterium tuberculosis (Mtb) results in the presence of EVs in the blood and secretions that carry Mtb fragments, specific proteins, and other substances, and there is hope that the contents within them can be used as biomarkers of tuberculosis or targets for drug guidance (9). As a result, the role of EVs in the diagnosis and treatment of tuberculosis, along with their associated clinical significance, is increasingly being accepted and recognized by the public. Research on using EVs as a new diagnostic method and treatment strategy for renal tuberculosis is receiving growing attention from the academic community.

The contents of EVs are highly complex, and extracting valuable biomarkers for the diagnosis and treatment of renal tuberculosis from them has been a significant challenge in recent research. Fortunately, studies have found that Lipoarabinomannan (LAM) and Lipoprotein G (LprG) are believed to have great potential. However, these two biomarkers differ in their biological functions and spatial structures, leading to differences in their roles and research value.

In this review, we will discuss recent literature on the changes in EVs secreted by hosts after Mycobacterium tuberculosis infection and summarize the potential and value of lipoarabinomannan (LAM) and lipoprotein (LprG) as new diagnostic markers and therapeutic targets for renal tuberculosis, hoping to provide new insights and references for future researchers in this field.

## 2 Lipoarabinomannan and Lipoprotein G in the bacterial extracellular vesicles of Mycobacterium tuberculosis

With the advancement of research on electric vehicles (EVs) in recent years, bacterial extracellular vesicles (bEVs) have garnered widespread attention from researchers. Depending on the bacterial species and the varying compositions of proteins, lipids, DNA, and RNA within bEVs, these bEVs often exhibit distinct biological activities. Zhao et al. summarized the characteristics of bEVs produced by known pathogens, including their ability to promote infection, protect the host, alter antibiotic or phage effects, regulate host immune function, and modulate cell proliferation (10, 11). In recent studies, EVs secreted by Mycobacterium tuberculosis have also been found to possess some of the characteristics above. Emilie et al.'s research on the components of bEVs secreted by Mycobacterium tuberculosis revealed that Mtb envelope lipoproteins, lipoglycans, and lipids exist in specific bEVs and can better reflect the characteristics of Mtb (12). Carolina et al.'s further research on bEVs secreted by M. tuberculosis (Mtb) found that the substances enclosed in these bEVs can regulate CD4+ T cells, which are then transferred to T cells to inhibit T cell responses, thereby allowing Mtb to evade the host's immune response. Moreover, LAM and LprG, substances present in these bEVs, are highly representative (13). Therefore, we speculate that M. tuberculosis bEVs carrying LAM and LprG may possess unique value.

### 2.1 Structure and spatial relationship of LAM and LprG

#### 2.1.1 LprG

LprG is a relatively conserved lipoprotein found in all mycobacteria and is an essential component for maintaining the virulence of Mtb. In conditions of limited nutrients, the loss of LprG function results in delayed maturation of Mtb. In the study by Fukushi et al., it was demonstrated that LprG suppresses inflammation in macrophages by downregulating nitric oxide (NO), cyclooxygenase-2 (COX-2), inducible nitric oxide synthase (iNOS), and pro-inflammatory cytokines through the nuclear factor κB (NF-κB), activator protein-1 (AP-1), and mitogen-activated protein kinase (MAPK) signaling pathways (14). The research design by Mariana et al. demonstrated at the genetic level that the presence or absence of LprG-related expression genes can influence the survival cycle of M. Tuberculosis in host cells (15). Therefore, the role of LprG in bEVs may be related to M. tuberculosis, as it reduces the host immune response and inflammatory reaction, thereby creating a more favorable cellular microenvironment. Additionally, LprG exhibits good stability even under certain extreme conditions, with minimal degradation by bacterial cells or reduction in its translation level (16, 17), suggesting its potential as a promising diagnostic marker for tuberculosis.

#### 2.1.2 LAM

LAM is a type of arabinose carbohydrate found on the extracellular vesicle membranes of Mtb, accounting for about 15% of the weight of Mtb (18). It has a core of D-arabinan, an enantiomer containing L-arabinose (19), which is anchored to the cell membrane by phosphatidylinositol (PI). Due to its ability to be separated together with lipoproteins and its good surface exposure characteristics, LAM is also one of the essential pathotoxicological molecules of Mtb (20, 21). Mtb can alter the activity of LAM through various modifications (including acylation, phosphorylation, and nitrogen modification), enhancing or reducing its infectivity and ability to maintain cellular stability (22). Among these modifications, succinylation is relatively common, and related studies have shown that this feature is associated with Mtb's involvement in inflammation (22).

Additionally, due to the presence of 5-methylthio-D-xylose (MTX) sugar on LAM, as well as a D-mannose (Man) cap structure encoded and co-regulated by bacterial genes such as Rv1635c and Rv2181, LAM exhibits specific antioxidant properties (23–25). Meanwhile, the chemical composition of LAM varies among different Mtb strains. The fact that LAM is found in the serum of tuberculosis patients suggests that it may affect multiple host cell populations, thereby influencing a range of biological processes of Mtb within the host (216). Sparks et al. designed an experiment using gene knockout. They summarized previous experimental results, demonstrating that LAM has a certain degree of value in the growth of M. tuberculosis and in maintaining bacterial envelope integrity. They hypothesized that LAM could regulate the activity of septal hydrolase. Still, when they knocked down the septal hydrolase gene RipA, it did not alleviate the cell shape defects of LAM-deficient mycobacteria, thus negating this hypothesis (26). The presence of LAM on the surface of M. Tuberculosis allows it to mimic specific glycoproteins on the surface of human or other mammalian cells. Moreover, these mannose family substances on the cell wall of Mtb, including LAM, may also play a role in mitigating the host immune response. Therefore, LAM has a certain degree of value in the infection of host cells by Mtb and the maintenance of the stability of Mtb cell structure. Additionally, due to its location on the cell wall, it is more conducive to recognition by the human immune system and the elicitation of a series of immune responses, as well as to secretion outside the cell when included in bEVs.

**Figure 1:**
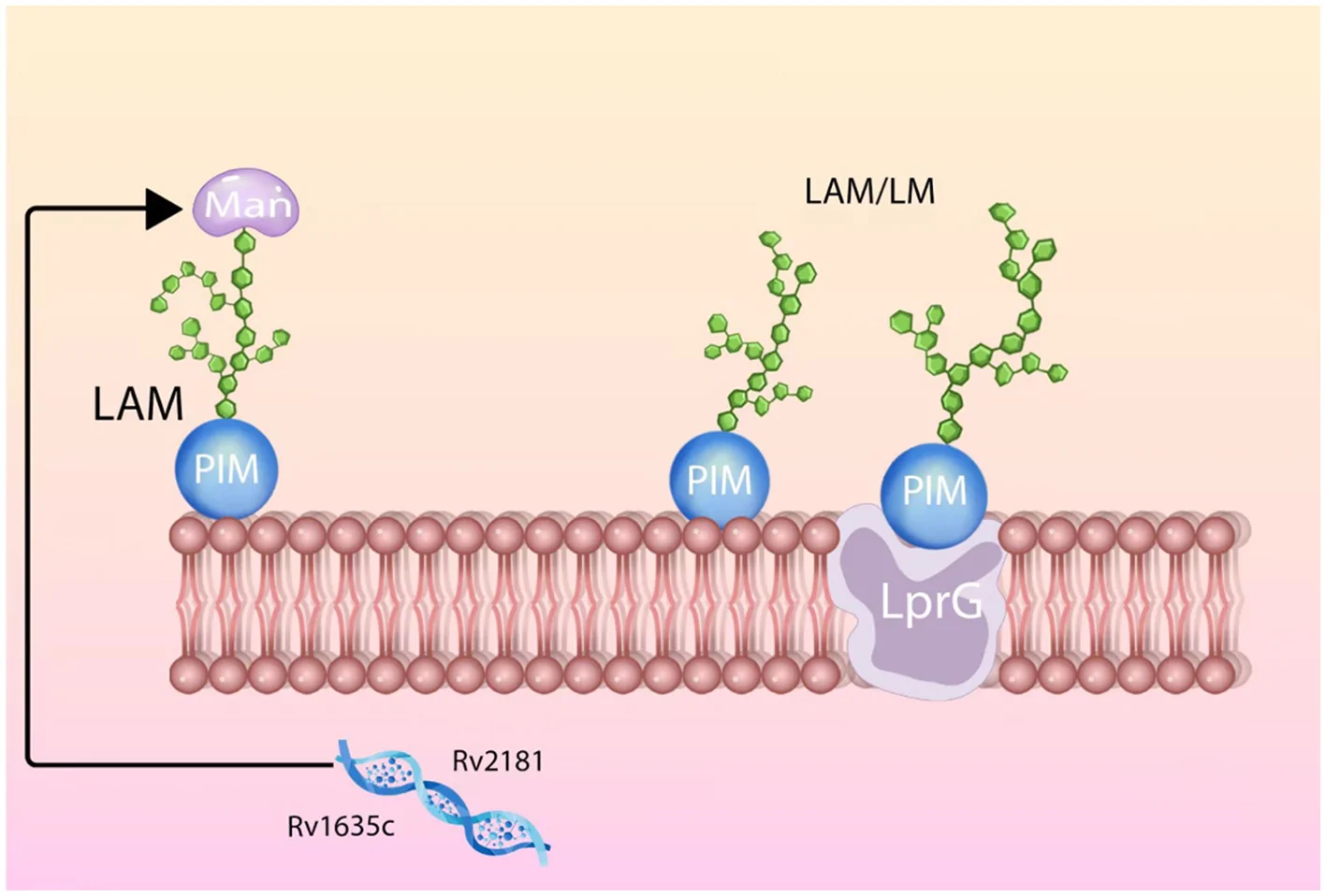
Diagram of the structure of LAM and LprG and their interrelationship.

Research has found that LAM and LprG have a very close spatial relationship. The hydrophobic pocket on LprG will interact with various types of LAM through acyl chains, and the mannose chains within LAM will also actively select to bind with LprG. This mutual selection binding mode not only stabilizes LAM on the cell membrane of Mtb but also facilitates the biological functions of LAM (27). Since LAM and LprG often co-occur, using LprG to localize LAM or identifying alternative methods to target LAM for diagnostic and therapeutic purposes has become a topic of interest. Based on the aforementioned related research, we have created a schematic diagram illustrating the spatial structural relationship between LAM and LprG, aiming to provide a visual explanation of this relationship (Figure 1).

Subsequently, a research team has publicly shared some vesicle proteins in the EVs secreted by Mtb through proteomic analysis (28) and summarized the currently identified protein composition in mycobacterial extracellular vesicles (29). It is foreseeable that more substances in the bEVs of Mtb will be discovered in the future. In recent years, several substances beyond LAM and LprG have been further confirmed to be related to the biological functions of Mtb in the host, including Cfp10, Cfp2, Mpt32, Mpt64, BfrB, LPqH, and LprA (13, 30–32). However, due to the properties of LAM and LprG, such as their resistance to mutation, good stability, and close spatial structure relationship, they hold greater significance in the diagnosis and treatment of tuberculosis.

### 2.2 LAM's immune response and cell signaling pathways

During the process of Mtb infection, LAM can regulate the host's immune response and infection progression through various cellular pathways. LAM primarily functions as an agonist of Toll-like receptor 2 (TLR2), directly or indirectly exerting its biological functions. It induces host cells to mount an immune response and produce inflammation that is favorable for the growth and reproduction of Mycobacterium tuberculosis. Through extensive summarization, we speculate that LAM plays a unique role throughout the entire process of Mycobacterium tuberculosis infection. The following are the main cellular pathways in which LAM is directly or indirectly involved, along with their respective roles.

#### 2.2.1 Inflammatory activation and regulation of immune response

##### 2.2.1.1 TLR2/MyD88/NF-κB pathway

The TLR2/MyD88/NF-κB pathway is a key inflammatory signaling pathway in the innate immune system, primarily involved in recognizing pathogen-associated molecular patterns (PAMPs) and damage-associated molecular patterns (DAMPs), thereby triggering inflammatory responses and regulating the immune system (33–35). Its core functions include activation of inflammatory responses, immune escape, and immune damage. The pathway is primarily initiated by TLR2 through the recognition of exogenous signals, including bacterial lipoproteins and certain fungal components (such as Aspergillus β-glucan), as well as endogenous signals (such as HMGB1), to initiate signal transduction. After activation by ligand binding, the TIR domain of TLR2 undergoes a conformational change, recruiting Myeloid Differentiation Factor 88 (MyD88) to form a complex, thereby initiating a series of downstream molecular effects (36–40).

As early as 20 years ago, Richard et al. demonstrated through relevant research that LAM is an agonist of TLR2 and can exert its biological effects by activating the TLR2/NF-κB pathway (41). In recent years, with the advancement of related research, we have learned that LAM can activate the Toll-like receptor 2 of cells, thereby activating the downstream MyD88-dependent pathway, triggering the nuclear translocation of NF-κB, and inducing the expression of immune response gene 1 (Irg1) to promote the release of cellular inflammatory mediators, such as interleukin-2 (IL-2), interleukin-4 (IL-4), interleukin-6 (IL-6), interleukin-10 (IL-10), interleukin-17A (IL-17A), tumor necrosis factor α (TNF-α), and granulocyte-macrophage colony-stimulating factor (GM-CSF), etc (42–45). LprG binds to TLR2 in the absence of TLR1, together with LAM and its precursors, but does not bind to TLR1 in the absence of TLR2 (218). At the same time, Mycobacterium tuberculosis can further modify LAM [e.g. by regulating protein O-mannosylation levels (220)] to inhibit the overactivation of TLR2 and further suppress the activation of related immune cells, such as T cells, thereby exerting its immune regulatory mechanisms to escape immune clearance (12, 46).

Furthermore, LAM is also released from Mycobacterium tuberculosis and can be detected in the intracellular or extracellular vesicles of infected cells. Therefore, the immunomodulatory properties of LAM are not limited to intact Mycobacterium tuberculosis but also apply to circulating LAM released in extracellular vesicles or apoptotic vesicles. This is the primary mechanism by which LAM exerts its immunomodulatory properties on infected or other cells (47).

The TLR2/MyD88/NF-κB pathway is a key link between innate immunity and inflammatory pathology. The type of ligand mainly influences the biological effects of its activation, but it primarily promotes inflammation and indirectly promotes fibrosis. Targeted therapy for key nodes in this pathway may provide new ideas and directions for the treatment of renal tuberculosis.

##### 2.2.1.2 Mannose receptor (MR) pathway

The mechanism of the mannose receptor (MR) pathway in renal tuberculosis involves complex interactions in pathogen recognition, immune regulation, and host defense. However, the primary biological function of the MR pathway is to utilize a class of type II transmembrane glycoproteins primarily expressed on the surface of immune cells such as macrophages and dendritic cells. These glycoproteins recognize glycosylated structures (such as mannose) on the surface of pathogens through their extracellular domains, mediating phagocytosis and promoting the formation of phagosomes (48–52).

MR is an efficient endocytic receptor, most notably characterized by multiple C-type lectin domains (CTLDs) within a single peptide chain. Specifically, the CTLD4 domain can recognize specific components, thereby mediating cellular immune regulation (213).

LAM on Mtb and its EVs can bind to mannose receptors (such as CD206) on the surface of macrophages through its mannose structure, thereby facilitating the phagocytosis of Mtb by macrophages and its entry into the cell (53, 54). However, due to the unique immunomodulatory and immune evasion functions of various components, including LAM, on the Mtb membrane, MR-mediated phagocytosis can accelerate the infection of host cells by Mtb. These immunomodulatory and immune evasion functions include inhibiting host DNA repair, reducing the activation of reduced nicotinamide adenine dinucleotide phosphate (NADPH) oxidase and phagosome acidification, blocking the maturation of macrophage lysosomes, and aiding the survival of Mtb within phagosomes; inhibiting the expression of major histocompatibility complex (MHC) class I and II molecules, thereby reducing antigen presentation; binding to TLR2 to modulate the release of inflammatory cytokines and enhance Th2-type immune responses (55, 56). According to relevant studies, activation of the MR pathway can affect T cell activation response and create conditions for Mycobacterium tuberculosis survival in host cells by regulating TLR4 signal transduction. However, the interaction mechanism between MR and TLR4 remains unclear (212).

This leads to MR-mediated immune phagocytic function, which will facilitate the settlement and reproduction of Mtb within host cells. Therefore, using drugs or other means to inhibit the binding of MR and LAM or promote the secretion of other immune factors to enhance the antigen-presenting ability, immune response effect, and phagosome function of host cells can be used as an alternative adjuvant therapy for renal tuberculosis.

##### 2.2.1.3 Mitogen-activated protein kinase (MAPK) pathway

MAPK pathway is a crucial intracellular signaling pathway. Although the composition of this pathway is complex, it is typically composed of several key cellular kinases or factors that form the central part of the pathway. These include extracellular signal-regulated kinases (ERK), c-Jun N-terminal kinases (JNK), and p38 mitogen-activated protein kinases (p38 MAPK). These members play critical roles in cellular signal transduction within the pathway, thereby regulating various physiological processes (57–60). Among these, ERK-mediated reactions are primarily involved in the transmission of signals for cell growth and proliferation, while JNK-mediated responses play essential roles in regulating apoptosis. p38 MAPK is typically associated with the expression of inflammatory factors and immune regulation (61–67).

Studies have shown that ERK1/2 is a conserved serine/threonine kinase, and its direct or indirect interactions with the PI3K/Akt, β-catenin, and Janus Kinase/Signal Transducer and Activator of Transcription (Jak/Stat) signaling pathways collectively mediate cell growth, regeneration, and proliferation. The activation of p38 MAPK can mediate the activation of NF-κB, thereby promoting the transcription and expression of inflammatory factors such as IL-6 and TNF-α, which in turn induces the organism's immune inflammatory response (68–70). Additionally, the JNK/MAPK pathway is closely linked to the apoptosis and autophagy processes of macrophages infected with Mycobacterium tuberculosis, and the activation of this pathway directly affects the host cell's immune defense capability against Mycobacterium tuberculosis (71).

LAM, a typical virulence factor of Mycobacterium tuberculosis, can mediate the MAPK pathway and induce the expression of IL-10 and IL-12 through the differential activation of p38 MAPK and ERK1/2, thereby synergistically regulating the inflammatory microenvironment of host cells in conjunction with TLR2 (44, 72). At the same time, although there have been no relevant studies in the past 5 years, based on pertinent previous studies, we speculate that LAM may mediate the phosphorylation of p47phox through the MAPK pathway and its related pathway crosstalk, thereby interfering with the activity of the NADPH oxidase complex and iNOS, reducing the production of reactive oxygen species (ROS) and nitric oxide (NO) (73–76). Although the effect of LAM on p47phond and whether it can affect p47phond through the MAPK pathway requires further research, the above studies still indicate that LAM can further reduce the bactericidal ability of macrophages and promote the survival of Mycobacterium tuberculosis by mediating the MAPK pathway.

##### 2.2.1.4 JAK/STAT3 pathway

The JAK/STAT3 pathway is a classic example of cellular signal transduction. Current research suggests that the activation of this pathway is linked to significant biochemical processes, including inflammatory responses, lipid metabolism, cell autophagy, and the progression of fibrosis (77–81).

Existing research suggests that LAM can promote the secretion of IL-10 through the JAK/STAT3 pathway while simultaneously inhibiting the production of IL-12, resulting in cytokine polarization, a weakened Th1 immune response, and an enhanced Th2 immune response, thereby affecting the Th1/Th2 balance (82, 83). Since the Th1 immune response is a crucial component of the anti-tuberculosis immune response, inhibiting this biological effect will lead to an imbalance in the host's immune response during chronic tuberculosis infection.

On the other hand, after LAM activates the signal transducer and activator of transcription (STAT3), it induces a suppressor of cytokine signaling 3 (SOCS3), thereby altering the activity and function of macrophages (84). Combined with its differential effects on IL-10 and IL-12, this further influences the formation or function of various cytokines and chemokines in macrophages (85), regulating the balance between the host's pro-inflammatory and anti-inflammatory responses.

##### 2.2.1.5 Mannose-binding lectin (MBL) pathway

The MBL pathway is a complement activation pathway that is triggered when foreign pathogens attack the body. It is typically activated by mannose and its analogs carried by pathogens, which bind to lectin receptors (such as the C-type lectin receptor Dectin-2) to activate complement pattern recognition molecules (PRMs), thereby activating this pathway (86–88).

Under conditions of Mtb infection, LAM can activate the complement system through the MBL pathway, making Mycobacterium tuberculosis highly susceptible to complement-mediated attack (89, 90). However, the Man cap and other structures on LAM can inhibit the formation of the complement membrane attack complex (MAC), and the sensitivity of LAM from different strains to receptors and the recognition of LAM by receptors vary, allowing Mycobacterium tuberculosis to evade complement-mediated lysis to a certain extent (91, 92).

The activation of the MBL pathway mediated by LAM can, to some extent, play a role in resisting Mtb's infection. Still, due to differences in receptor recognition, the MBL pathway often exhibits a certain activation lag, and LAM can regulate the inflammatory response through other pathways, thereby interfering with the progression of the immune response. Therefore, the complement immune function of this pathway may not be able to resist the invasion of Mtb fully but instead may induce the diseased tissue to enter a state of chronic infection.

#### 2.2.2 Regulation of apoptosis and autophagy

##### 2.2.2.1 Inhibition of apoptosis—PI3K/Akt pathway

Previous studies have shown that the virulence factor LAM of Mycobacterium tuberculosis can inhibit T cell migration to lymphoid tissues by interfering with the PI3K/AKT pathway, thereby reducing the initiation of Th1 effector responses in diseased tissues, leading to chronic infection (93). It can also interfere with calmodulin-dependent phosphatidylinositol 3-phosphate (PI3P) production, preventing phagosome maturation and thus ensuring the survival of Mycobacterium tuberculosis within the phagosome (94). These findings suggest that LAM can modulate the inflammatory response and enhance bacterial survival through the PI3K/Akt pathway.

As research teams explore the PI3K/Akt pathway, it has been found that LAM can not only induce infection and prevent phagosome maturation through the PI3K/Akt pathway but also regulate macrophage apoptosis infected with Mycobacterium tuberculosis and promote macrophage survival by promoting the phosphorylation of Bad, a pro-apoptotic protein in the B-cell lymphoma-2 (Bcl-2) family; regulating the activation of Caspase-3; and increasing the expression of the anti-apoptotic protein B-cell large lymphoma protein (Bcl-xL) (95–97).

This allows Mycobacterium tuberculosis to survive for a prolonged period in macrophages, providing a relatively safe breeding ground for the bacterium. However, in recent years, there have been few further studies on the regulation of the PI3K/Akt pathway by LAM, and additional research is still needed to explore whether the path mediated by LAM can affect macrophage apoptosis by modulating other cytokines and influence host apoptosis by interacting with other cellular pathways.

##### 2.2.2.2 Inhibition of autophagy—mammalian target of rapamycin (mTOR) pathway

Recent studies have shown that LAM can inhibit the activation of the mTOR pathway and interfere with the downstream TCR-CD3, CD28, and interleukin-2R (IL-2R) signal transduction, thereby affecting the activation, proliferation, and interleukin-2 (IL-2) gene transcription and translation of T cells (especially CD4+ T cells), and further affecting the activation of T cells (98, 99).

At the same time, LAM can also inhibit the maturation of phagosomes in various ways by mediating the mTOR pathway. Although no relevant research reports have been seen in the past 5 years, we have summarized the appropriate research on mTOR and speculate that it may affect the function, growth, and morphology of phagosomes by inhibiting the downstream ULK1-Beclin1 molecules, thereby inhibiting autophagy (100–102). Further experiments are needed to elucidate the mechanisms by which LAM affects the mTOR pathway and other effects on phagosome formation, including how to target phagosome formation, how to interfere with phagosome function, and how to regulate downstream molecules.

LAM can modulate the autophagy process in all host cells, including macrophages, by interfering with the mTOR pathway and affecting the function of phagosomes, as well as the activation of T cells, thereby enabling Mtb to survive long-term within host cells.

#### 2.2.3 Angiogenesis and fibrosis

##### 2.2.3.1 Vascular formation and hyperplasia—vascular endothelial growth factor (VEGF)

VEGF is a cytokine that regulates vascular endothelial proliferation and is co-regulated or regulated by various upstream substances. During the prolonged course of Mtb infection, M. tuberculosis induces the aggregation of cytokines related to vascular proliferation and differentiation (such as IL-6, VEGF, and TNF-α) at the lesion site, thereby participating in the formation of granulomas, which are crucial in tuberculosis lesions, by promoting angiogenesis.

Current research suggests that LAM may increase vascular permeability through the TLR2/VEGF pathway, and the degree of vascular permeability increase is positively correlated with VEGF (103) and may promote angiogenesis through the HIF-1α/VEGF pathway directly or indirectly (104), which may indicate a close relationship between LAM and granuloma formation in renal tuberculosis. At the same time, due to the increase in VEGF caused by Mycobacterium tuberculosis, the newly formed blood vessels are unable to enhance local tissue blood supply, resulting in poor wound healing after tuberculosis resection (105).

Based on this connection, current research suggests that VEGF and its related pro-angiogenic cytokines can differentiate between latent and active Mycobacterium tuberculosis infection (106–108). In the future, we can explore whether LAM can also determine this infection by further studying the correlation between LAM and VEGF.

##### 2.2.3.2 Fibrosis—transforming growth factor-β (TGF-β)

Fibrosis is a key feature of Mycobacterium tuberculosis infection. Current research has found that the levels of transforming growth factor-β (TGF-β), IL-10, matrix metalloproteinase-1, and other substances in the blood of tuberculosis patients are elevated. These substances mediate the progression of fibrosis at the lesion site (109, 110) and contribute to the formation of chronic inflammatory granulomas within the lesion (111).

Although LAM cannot directly activate TGF-β, we believe that LAM may indirectly activate the TGF-β/Smad pathway during long-term infection, thereby contributing to the tissue fibrosis process to a certain extent (for example, by activating TLR2 and utilizing its downstream molecules to influence the function of the TGF-β/Smad pathway) (112). The activation of TGF-β may be associated with the destruction of renal parenchyma and the development of ureteral stenosis in renal tuberculosis.

Current research has shown that TGF-β can be targeted by drugs to combat Mycobacterium tuberculosis infection (113). Therefore, we have reason to believe that regulating TGF-β levels may be a promising direction for future anti-tuberculosis fibrosis treatment (114).

**Figure 2:**
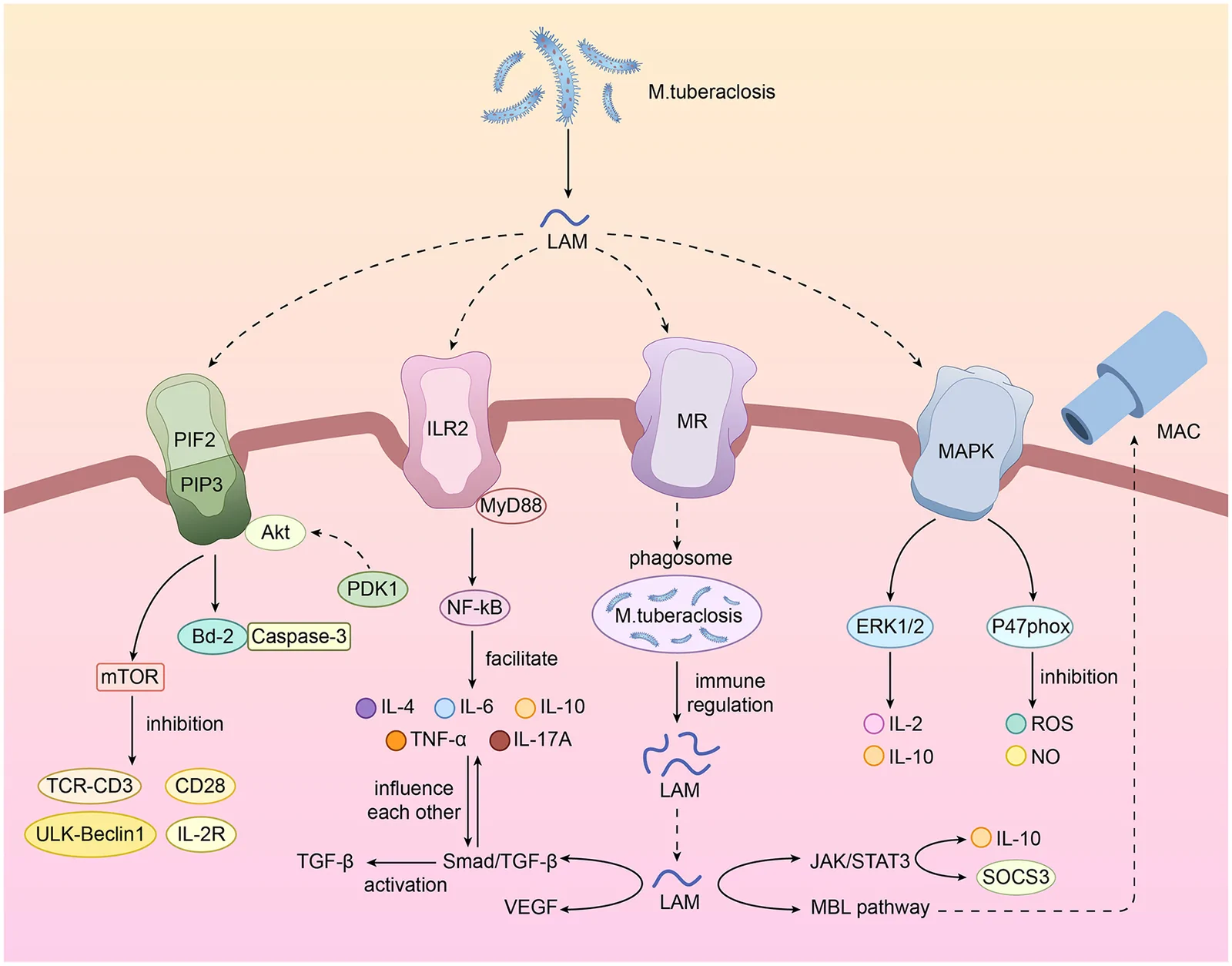
Cellular signal transduction pathways and functions of LAM.

In summary, LAM plays a dual role in the cellular signaling pathways of renal tuberculosis. On the one hand, LAM can mediate immunity and enhance bacterial survival through pathways such as TLR2, the mannose receptor, the MAPK pathway, the MBL pathway, and the inhibition of apoptosis and autophagy. Secondly, LAM may cause damage to the renal structure by inducing angiogenesis (VEGF) and promoting fibrosis (TGF-β) directly or indirectly. We have compiled a summary table and drawn a relevant pathway diagram for quick reference (Table 1; Figure 2) of the cellular signaling pathways affected by LAM during the pathological process of renal tuberculosis. Further research on the cellular signaling pathways of LAM will help us better understand the entire pathophysiological process of renal tuberculosis and even urinary system tuberculosis.

**Table 1:** Cell signaling pathways directly or incidentally related to LAM and their biological functions.

**Biological effect**	**Cell signaling pathways related to LAM**
Inflammatory activation and regulation of immune response	TLR2/MyD88/NF-κB pathway
	MR pathway
	MAPK pathway
	JAK/STAT3 pathway
	MBL pathway
Regulation of apoptosis and autophagy	PI3K/Akt pathway
	mTOR pathway
Angiogenesis and fibrosis	VEGF
	TGF-β

### 2.3 LprG's immune response and cell signaling pathways

During the process of Mtb's infection, LprG can influence the pathogenicity and drug resistance of Mtb by regulating cell wall permeability and inducing host immune evasion mechanisms. Our review of relevant studies has found that the relationship between LprG and LAM is more synergistic, where LprG facilitates the biological functions of LAM and further induces immunity, as well as maintains cell wall stability. The following describes the biological roles of LprG in immunity and its associated cellular signaling pathways.

#### 2.3.1 Regulation of cell wall permeability

Previous studies have demonstrated that LprG is a crucial lipoprotein of Mycobacterium tuberculosis, which can bind to lipoarabinomannan (LAM) and facilitate the transport of LAM from the cytoplasm to the bacterial surface, thereby exerting its virulence functions. Additionally, LprG maintains the integrity of the cell wall through its role as a lipoprotein anchor (115).

Deletion or mutation of LprG can alter cell wall permeability, leading to changes in the sensitivity of bacteria to anti-tuberculosis drugs or anti-infective agents such as vancomycin and ethambutol (116, 117). Subsequent experiments with ethidium bromide (EtBr) permeability confirmed that the integrity of the cell membrane in LprG mutant strains was compromised, allowing drugs to enter the bacterial interior more easily (15) and making Mycobacterium tuberculosis more susceptible to killing by drugs and the host immune system.

Therefore, LprG can serve as a new target for anti-tuberculosis drugs, and its inhibitors, in combination with existing drugs, can enhance the bactericidal effect and shorten the treatment cycle (118).

#### 2.3.2 Synergistic effects of LAM on host immune response

In the preceding text, we mentioned that LprG can influence the production of cytokines and pro-inflammatory mediators through various signaling pathways, such as the TLR2/NF-κB pathway, thereby modulating the host inflammatory response (14). The impact of LprG on immune and cellular signaling pathways primarily involves enhancing the function of LAM, facilitating its better release, and exerting its role in regulating inflammation, immune evasion, and immune homeostasis. This auxiliary and synergistic effect of LprG may promote the long-term latent infection of Mtb in the urinary system.

#### 2.3.3 Auxiliary regulation of lipid metabolism

Lipid synthesis, accumulation, and subcellular distribution are crucial for the cellular homeostasis of Mycobacterium tuberculosis, and lipid metabolism is closely related to bacterial growth rate regulation, virulence, and antibiotic tolerance (211). LprG, together with Rv1410c from the same operon, can jointly regulate the transport and metabolism of lipids (especially triglycerides), which are essential sources for the synthesis of Mtb's cell wall components. It is important to note that LprG is anchored in the outer leaflet of the cytoplasmic membrane through three acyl chains. At the same time, Rv1410c functions to extract LprG from the membrane, allowing it to cross the cytoplasm and reach the outer membrane to transport lipids. Therefore, the absence of LprG may lead to disorders in lipid metabolism and transport, affecting the stability of the cell wall lipid layer and thereby increasing the sensitivity of bacteria to drugs (119–122). Drugs targeting the LprG-regulated lipid metabolism pathway may disrupt the cell wall structure of Mycobacterium tuberculosis, thereby enhancing the efficacy of existing drugs (123).

**Figure 3:**
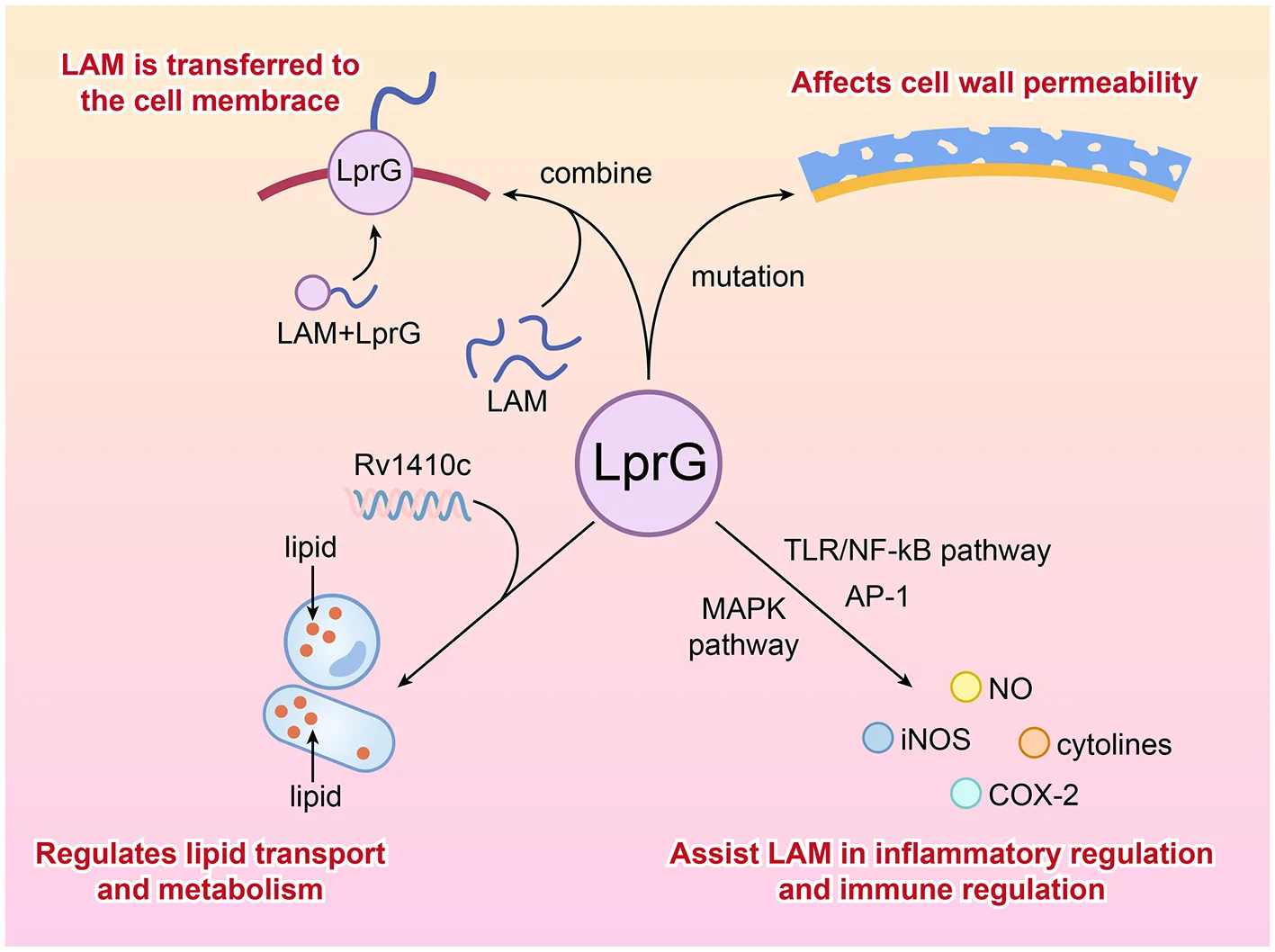
Cellular signal transduction pathways and functions of LprG.

In summary, LprG primarily plays a role in Mycobacterium tuberculosis in assisting LAM in regulating immunity, lipid metabolism, and stabilizing the cell wall. The absence or inhibition of LprG can enhance the permeability of anti-tuberculosis drugs and improve the bactericidal effect. LprG can assist LAM in regulating the inflammatory response through the TLR2/NF-κB pathway, promoting the immune evasion of Mycobacterium tuberculosis. It also affects lipid metabolism and synergizes with LAM to enhance the pathogenicity of Mycobacterium tuberculosis. We have summarized the above content and created a summary table for reference (Table 2; Figure 3).

**Table 2:** Characteristics and biological functions of LprG.

**Characteristics of LprG**	**Biological functions**
Assist in mediating the TLR2/NF-κB pathway	Adjuvant LAM affects the production of cytokines and pro-inflammatory mediators.
The linking role of lipoproteins	Maintain cell wall stability
Binding to LAM	Promote the translocation of LAM to the cell membrane
Synergy with Rv1410c	Regulate lipid transport and metabolism

## 3 The value of LprG in the diagnosis of renal tuberculosis

In the pathophysiological process of various types of tuberculosis, the relationship between LprG and LAM is inseparable. However, in terms of diagnostic value, since LprG is not considered a major virulence factor of M. Tuberculosis, it currently primarily serves as a substance to aid in improving LAM detection. In patients with renal tuberculosis, LprG, as a relatively fixed component of M. Tuberculosis's extracellular vesicles (124), can be used to target and enrich the EVs secreted by M. Tuberculosis, thereby improving the detection rate of LAM. Meanwhile, due to its low toxicity, LprG has advantages over LAM in vaccine preparation (125, 126).

In some special populations, such as HIV patients and children, due to immune function defects or incomplete development, it is challenging to detect tuberculosis using conventional methods. In a study on the diagnostic efficacy of LprG and LAM in pediatric tuberculosis patients, LprG plays an important role in anchoring LAM to the cell membrane of Mtb and serves as a carrier protein for LAM. Therefore, using LprG for EV capture and LAM detection has the potential to become an emerging diagnostic technique for tuberculosis. In this experiment, researchers tested the levels of LprG and LAM in various samples, including urine samples closely related to the diagnosis of renal tuberculosis. The results showed that even in urine samples that had been repeatedly decomposed and filtered, both markers had higher detection rates for tuberculosis compared to the Xpert method. Moreover, since this detection method can use non-sputum samples, its detection rate for extrapulmonary tuberculosis, including renal tuberculosis, is also excellent (127).

To date, no studies have analyzed the potential of the single LprG molecule in the diagnosis of tuberculosis. Therefore, further in-depth research is still needed to investigate the role of single LprG in the diagnosis of both pulmonary and extrapulmonary tuberculosis.

## 4 The value of LAM in the diagnosis of renal tuberculosis and the exploration of novel LAM detection methods

### 4.1. New LAM detection methods and their significance in clinical practice

In a meta-analysis of HIV-infected individuals, researchers analyzed 844 records from 20 datasets and 10,202 participants, finding that Alere LAM had a higher detection rate among subjects with low CD4 counts and hospitalized patients. Alere LAM offers a rapid turnaround time and simplicity. Because almost all participants can provide urine samples, it has an advantage over Xpert in utilizing non-sputum specimens, regardless of patient symptoms, the presence or absence of sputum, or CD4 cell count (128, 129). Additionally, due to its higher sensitivity in diagnosing renal tuberculosis and ease of sample collection, Alere LAM is more acceptable to patient populations compared to Xpert. Its lower economic cost provides a unique advantage over invasive and expensive pathological and immunological tests. In another study of malnourished children and the use of LF-LAM for tuberculosis detection in HIV adult patients, LAM also showed promising detection rates (130, 131). In an interview study conducted in Ghana using Determine LAM, researchers found that despite some healthcare workers' concerns about confidentiality during the actual testing process, the quick and straightforward testing procedure and easy sample collection did not hinder patient diagnosis and treatment. Another prospective observational study using Determine LAM for tuberculosis detection in critically ill patients also showed that LAM testing maintained reasonable specificity even in severe cases (132). Therefore, selective use of LAM testing in critically ill patients can improve diagnostic rates to some extent (133).

Due to the fact that LAM testing can utilize urine samples, which are less constrained by time and environmental factors compared to blood or other body fluid samples (134), LAM determination is expected to replace Xpert as a new diagnostic criterion or be used in combination with other diagnostic markers for renal tuberculosis, especially in special populations and patients with severe diseases and complications (135, 136). With continuous technological advancements, emerging LAM detection methods have been successively introduced, offering improved detection rates and sensitivity, which has gradually gained public recognition and acceptance in the field of pulmonary and extrapulmonary diagnosis (137–139).

In recent years, research teams have attempted to explore the advantages and disadvantages of these novel or standard LAM detection methods in the diagnosis of pulmonary tuberculosis or extrapulmonary tuberculosis. Paul et al. conducted experiments to investigate the effect of LAM content in various samples on detection rates. The results showed that urine samples had a sensitivity of 62%, while serum or plasma samples had a sensitivity of 70% (140). The research team, led by Getachew et al., employed a systematic review and meta-analysis approach to assess the diagnostic value of the Alere LAM test, MTB-LAM-ELISA, and Fuji LAM test for pediatric tuberculosis. The results indicated that the sensitivity and specificity of the Alere LAM test were lower than those of the Fuji LAM test.

In comparison, the MTB-LAM-ELISA test exhibited higher specificity but lower sensitivity (141). Another prospective multicenter study and recent research on the Fuji LAM test for pediatric tuberculosis have further confirmed the test's better sensitivity and specificity (137, 138, 142, 143). Regarding the accuracy and cost-effectiveness of the Fuji LAM test, existing studies have demonstrated that its sensitivity is influenced by various factors, including batch number, sample size, and the number of CD4 (+) T cells, and that it can be used concurrently with the WHO-endorsed Xpert Ultra to reduce diagnostic costs for patients (144, 145).

Zhang et al. conducted a prospective observational study using a combined detection method of urine LAM testing (chemiluminescence method) and GeneXpert in HIV-infected tuberculosis patients. The results showed that the combination of Fuji LAM and GeneXpert methods could significantly improve detection rates (146). Another study incorporated bacterial culture testing and samples from extrapulmonary tuberculosis patients using this method, also achieving reasonable detection rates (147). A study conducted in Ethiopia demonstrated that the sensitivity of combined Xpert MTB/RIF and TB-LAM diagnostics for patients with extrapulmonary tuberculosis (EPTB) is superior to that of single tests (148). This suggests that the detection rate of LAM testing when combined with other diagnostic methods, may be superior to LAM testing alone.

A trial using molecular bacterial load assays for Mycobacterium tuberculosis in urine samples from HIV-infected patients with tuberculosis showed that the use of Mycobacterium tuberculosis testing could improve the diagnostic efficiency of tuberculosis as a supplementary test (149). Another study on oral swab PCR+LAM assays for the detection of tuberculosis in HIV patients also suggested that tongue swabs can be used as a supplementary test for LAM assays and other tuberculosis tests (150). The conclusions of these studies indicate that the current standard LAM testing combined with other testing methods will help improve the detection rate of tuberculosis and extrapulmonary tuberculosis, especially for patients with special populations such as HIV. Unfortunately, it also appears that standard LAM testing methods alone do not yield significant clinical benefits.

However, this does not mean that the effectiveness of the Fuji LAM test in clinical diagnosis is not recognized. Some scholars believe that the diagnosis of renal tuberculosis may be achieved by combining the Fuji LAM test with other examination methods (134, 151–155). However, currently, there are numerous LAM determination instruments on the market, with varying detection efficiencies and times. Therefore, we summarized the existing LAM detection methods mentioned in the above studies and compared their advantages and disadvantages (Table 3). Selecting an appropriate LAM detection method based on clinical needs or developing a more accurate and straightforward LAM detection method can reduce the time it takes for patients to start treatment to some extent (156).

**Table 3:** Current diagnostic methods and advantages and disadvantages of renal tuberculosis through LAM.

**The method of diagnosing renal tuberculosis through LAM**	**Advantages**	**Disadvantages**
Alere LAM (based on lateral flow immunochromatography technology)	The method provides rapid results, simple operation, and easy sample acquisition (128, 129)	Samples need to be tested as soon as possible after collection; test strips need to be strictly stored at low temperatures; in non-HIV-infected populations, sensitivity is usually low (210)
LF-LAM (Based on immunochromatography technology)	Interpretation of results is intuitive; results are obtained very quickly; samples are easy to get (130)	The detection sensitivity of LF-LAM is relatively low in non-HIV populations (130, 131)
Determine LAM (based on lateral flow immunochromatography technology)	The sample collection and processing procedures are relatively simple, the results are obtained quickly, and the operation is simple (132)	In non-HIV-infected or immunocompetent patients, its sensitivity is significantly reduced (132)
MTB-LAM-ELISA (Based on enzyme-linked immunosorbent assay)	Low cost, suitable for large-scale screening, high reagent stability, and good test reproducibility (141)	[Low sensitivity; false negatives may occur in early-stage patients (141)]
TB-LAM (based on lateral flow immunochromatography technology)	Results are obtained extremely quickly; the operation process is straightforward (148)	Among non-HIV-infected populations, the sensitivity is relatively lower (148)
Oral swab PCR + LAM assay (Based on molecular biology techniques)	Both sensitivity and specificity are relatively ideal; the potential risk of infection for healthcare workers is low (150)	Detection cost is high; the procedure is relatively complex; reproducibility is poor (150)
Fuji LAM (Based on immunochromatography)	Good sensitivity and specificity (137, 138, 142, 143)	The detection rate is affected by objective factors such as sample limitations and product batch numbers (137, 143)

### 4.2 Improved method for LAM detection—proteinase K

Currently, research teams have proposed and validated a scheme to optimize LAM testing using proteinase K. Recent studies have demonstrated that deproteinization of LAM with proteinase K can reduce the steric hindrance effect of LAM, thereby significantly improving the detectability of LAM (157–159). Huang et al. developed a novel LAM detection method using proteinase K-pretreated concanavalin A (ConA) to bind to LAM in urine for ELISA detection, finding that this method improves the sensitivity of LAM detection (160). Christopher et al. reported an automated detection platform that uses proteinase K-pretreated samples to determine LAM, allowing for determination with a reduced sample volume (161). The biological action of proteinase K can undoubtedly enhance the interaction between LAM and labeled antibodies, antigen-antibody complexes, and sensors, thereby improving the detection rate of LAM in urine samples. Since patients with renal tuberculosis often have very few urine samples collected due to various complications such as nephrectomy in the late stage, and after pretreatment of samples with proteinase K, fewer samples can be used for detection, so pretreatment of LAM with proteinase K is one of the optional schemes for LAM determination in the future.

### 4.3 Improved method for LAM detection—design of a novel sensor

In the design of new LAM determination sensors, Dinesh's research team developed a sensor composed of a nanocomposite synthesized from multi-walled carbon nanotubes and zinc oxide (MWCNT-ZnO) to enhance the binding efficiency of LAM and antibodies (162). Ubaid et al. designed a sensor based on phase shift cavity ring-down spectroscopy (PS-CRDS) and activated the surface of the tapered fiber in the sensor with the anti-LAM antigen CS-35, resulting in considerable sensitivity even when the sample concentration was as low as 10 pg/mL (163). In addition, research reports have been published on the determination of LAM by designing a plasma biosensor (P-FAB), which can be used alone or in combination with gold nanoparticles (AuNP) to detect LAM, thereby achieving the goal of reducing the sample size required for determination. At the same time, its sensor probes and AuNPs are also easy to preserve (164, 165), which enhances their operability in actual clinical work. At the same time, the current popular machine learning can also be applied to this type of detection (166). The research on various new sensors makes the detection samples of LAM more accurate and traceable, and more nanosensors may be designed in the future to facilitate the early screening of extrapulmonary tuberculosis, such as renal tuberculosis.

### 4.4 Improvement of LAM detection method—application of nanoions

In recent years, the method of screening or detecting pathogens through nanoparticles has been evaluated as effective and highly stable by relevant experiments (167, 168). Meng et al. designed a portable instrument and used it to analyze urine samples from tuberculosis patients via copper ion probe, nano-ion cascade amplification, and stabilizer stabilization. The results showed 100% specificity and 90% sensitivity (169). Another experiment using copper nanoparticles to design a new LAM determination method also showed good diagnostic potential (170). This suggests that the improvement of LAM detection methods using nanoparticles can significantly enhance detection efficiency, which is of considerable clinical significance for diagnosing extrapulmonary tuberculosis with atypical clinical symptoms, such as renal tuberculosis.

### 4.5 Improvement of LAM detection method—design of new antibody

On the other hand, in the design and selection of new antibodies, due to the diversity of LAM antibodies, their ability to recognize and bind to LAM varies depending on the type of LAM antibody, and their impact on test results also varies (171–173). The PATHFAST TB LAM Ag assay is a new LAM assay designed based on chemiluminescent enzyme immunoassay. A research team has demonstrated through experiments that the sensitivity of this assay is 88.8%. The specificity is 100.0% (174), indicating that the PATHFAST TB LAM Ag assay has potential value for monitoring tuberculosis treatment and may be further applied in the field of extrapulmonary tuberculosis. Some research teams have developed or utilized high-affinity rabbit monoclonal antibodies to enhance detection methods, resulting in improved detection efficiency, whether through conventional ELISA experiments or other high-sensitivity detection methods (175–177). The use of reagents such as cyanobacterial lectin microvilli-N (MVN) to enhance the binding capacity between LAM and LAM antibodies in the design of magnetic bead ELISA experiments can also improve detection capabilities (178).

### 4.6 Summary of LAM detection methods

Detection methods based on ELISA for lipoarabinomannan (LAM) in extracellular vesicles have shown good diagnostic efficacy in pulmonary tuberculosis and extrapulmonary tuberculosis, including renal tuberculosis. Currently, numerous new LAM diagnostic methods are being developed. We have summarized these new and modified LAM diagnostic methods and their effects for reference (Table 4). It can be said that LAM detection is a landmark method in the diagnosis of tuberculosis. From the results, even in urine samples that have undergone multiple rounds of decomposition and filtration, any detection method for LAM in extracellular vesicles has a higher detection rate for tuberculosis than the Xpert method. However, current guidelines only recommend urine LAM testing for HIV patients who meet established criteria. Considering the superiority of LAM detection and other advantages, some research teams suggest expanding LAM testing to different populations, including non-HIV patients (179), or suggesting routine Fuji LAM testing for hospitalized HIV patients (180). This is particularly urgent in medical wards where patients often have multiple underlying diseases, and current treatment plans do not include specialized surgical treatments. Renal tuberculosis rarely shows clinical symptoms in the early stages or only manifests as common urinary tract infection symptoms. Moreover, HIV patients are not only more likely to develop renal tuberculosis, but their mortality rate is also higher (181). Therefore, LAM testing for HIV patients in medical wards can detect a large proportion of occult tuberculosis, such as renal tuberculosis or other urinary system tuberculosis patients, thereby further extending the patient's survival cycle.

**Table 4:** Novel or improved LAM detection methods and their effects.

**New or modified LAM detection methods**	**Modified effect**
Proteinase K	To reduce the steric hindrance effect of LAM and improve the detection rate of LAM (157–159)
Design of a novel sensor	A smaller sample size is required for measurement; the sensor is easy to store; AI technology can be applied to assist in the detection process (164–166)
Application of nanoions	Nanoions have good stability and can enhance the detection rate (167, 168)
Design of novel antibodies	The detection efficiency of LAM is higher (175, 177)

## 5 The value of LAM and LprG in extracellular vesicles in the treatment of renal tuberculosis

In the course of research on anti-tuberculosis drugs and tuberculosis prevention, numerous studies have suggested that different treatment strategies can be designed based on the characteristics of LAM and LprG in Mycobacterium tuberculosis, thereby assisting in anti-tuberculosis treatment or the design of vaccines or vaccine adjuvants. These treatment strategies include traditional Chinese medicine prescriptions, new drugs, cell pathway targeting, and immune enhancement, among others. These treatment strategies and vaccine designs have unique features.

### 5.1 Traditional Chinese medicine and novel synthetic drugs

Drug design targeting LAM and LprG is primarily achieved by interfering with their effects on cell signaling pathways or disrupting their synthesis. Current research has found that it is relatively feasible for researchers to develop inhibitors targeting these pathways, such as the mTOR inhibitor sirolimus, traditional Chinese medicine Scutellaria baicalensis, and Sophora flavescens preparations, by inhibiting the activation of signaling pathways and interfering with the metabolism and function of Mycobacterium tuberculosis (182). However, designing drugs to reduce the toxicity of Mycobacterium tuberculosis or to kill it by disrupting the synthesis of both is relatively more challenging.

#### 5.1.1 Inhibitioninhibition and immune regulation programs targeting TLR2-related pathways

Since LAM is an activator of TLR2, it can be used to treat tuberculosis by inhibiting the biological effects of this pathway. Current reports indicate that andrographolide can reduce inflammation by inhibiting the Notch1 pathway and down-regulating the phosphorylation of the NF-κB p65 subunit (183, 184). Other related substances in traditional Chinese medicine also have similar anti-inflammatory effects (185).

For TLR2 itself, current research techniques, such as gene silencing or TLR2 knockout, can improve inflammation and reduce vascular fibrosis to a certain extent (186). Transferring relevant research to the treatment of renal tuberculosis may be more conducive for clinicians to understand the role of TLR2 in the pathophysiology of renal tuberculosis.

#### 5.1.2 Inhibitioninhibition and immune regulation programs targeting the MR pathway

Because Mycobacterium tuberculosis accelerates its intracellular colonization through the MR-mediated phagocytic process of host cells, the use of mannose analogs can block the binding between pathogens and host cells, thereby reducing infection. Additionally, monoclonal antibodies targeting MR can inhibit IL-4/IL-13-induced Th2-type responses and reverse immune escape (187, 188).

Or by inhibiting its binding to the receptor, such as the glucose mimetic 2-deoxy-D-glucose (2-DG), thereby preventing LAM from binding to the receptor to inhibit the pathway (189); polysaccharide immunostimulants (such as Poria cocos polysaccharides) can interact with MR (mannose receptor), increase the secretion and mRNA expression of TNF-α within cells, and inhibit tuberculosis infection (190).

Current research suggests that the treatments mentioned above can be combined with classic anti-tuberculosis drugs to form an anti-tuberculosis drug + immunomodulator regimen (such as the standard anti-tuberculosis regimen (e.g., 2HRZE/4HR) combined with the traditional Chinese medicine Poria cocos), which can increase the MTB negative conversion rate and improve the CD4+/CD8+ ratio. Additionally, immune enhancement methods can be employed to enhance antigen presentation through nanoparticles, nano-vaccines, and other carriers or by utilizing the mannose selectivity of MR to fabricate corresponding mannose into microfilms and assemble other drugs, thereby achieving an auxiliary anti-tuberculosis effect (191–197).

#### 5.1.3 Inhibition of the mTOR pathway and immune modulation strategies

Recent studies have shown that baicalein, a compound found in the traditional Chinese medicine Scutellaria baicalensis, can inhibit the apoptosis of macrophages infected with Mycobacterium tuberculosis by suppressing the Akt/mTOR pathway, downregulating the assembly of absent in melanoma 2 (AIM2) and NOD-like receptor and pyrin domain-containing protein 3 (NLRP3) inflammasomes, and promoting autophagy, thereby making it difficult for Mycobacterium tuberculosis to spread further. Since LAM can act on the mTOR pathway, the inhibitory effect of baicalein on this pathway can also hinder the function of LAM (198).

#### 5.1.4 Inhibition of the MAPK pathway and immune modulation strategies

The emergence of various inflammatory diseases in humans is closely related to the dysregulation of the MAPK signaling pathway. Current research suggests that tuberculosis, a disease characterized by inflammation, can be treated and regulated by inhibiting the MAPK pathway (59). LAM, as one of the mediators involved in the MAPK pathway, can also be prevented from exerting its inflammatory regulatory effects due to the inhibition of this pathway.

In the study of related drugs, isoliquiritigenin from licorice has been shown to exert its anti-inflammatory effects by inhibiting the activation of the MAPK signaling pathway (199). A new synthetic drug, 1,2-ethylenediamine (SQ109), can also assist in the treatment of tuberculosis by mediating the MAPK pathway to stimulate M1 macrophage polarization and induce the production of protective pro-inflammatory cytokines (57).

Currently, the design of drugs that target the MAPK pathway for Inhibition to combat the functions of LAM and LprG, thereby assisting in anti-tuberculosis treatment, is a hot topic in recent years. In the future, more drugs may be developed, but MAPK pathway inhibitors will remain key. This is not only related to the characteristics of LAM and LprG but also to the impact of the MAPK pathway on inflammatory mediators.

#### 5.1.5 Inhibitioninhibition and drug design challenges for other potential pathways

LAM and LprG can have direct or indirect effects on various pathways within host cells. For the LAM-mediated MBL pathway, complement activators can be designed to enhance the role of complement immunity; however, there are currently few reports of similar drugs being used in clinical research for anti-tuberculosis drugs. In the field of regulating angiogenesis and mediating fibrosis, since LAM may only play an indirect role in these two pathophysiological processes, the treatment of these two pathological processes may not be achieved by a single inhibition of LAM and LprG, so there are also few similar research reports.

At present, while research on drugs that counter the functions of LAM and LprG and disrupt their synthesis is steadily progressing, it still faces many difficulties. For example, most studies remain at the level of *in vitro* experiments and lack *in vivo* verification. It is difficult to analyze the single-target effect of the synergistic action of multiple components in traditional Chinese medicine compounds, and the complex toxicology of Western medicine synthesis requires large-sample and multi-center clinical studies. At the same time, differences in the batches and purity of the raw materials used to manufacture medicinal drugs may affect their efficacy, among other factors. Therefore, there is still a lot of work to be done on the drug design of LAM and LprG.

Moreover, drug design targeting renal tuberculosis is not limited to LAM and LprG. Recent studies on bacterial urease C (UreC) have shown that urease inhibitors can disrupt the growth environment of Mtb and inhibit its reproduction by preventing UreC from interacting with RuvB-like protein 2 (RUVBL2) and impeding the formation of RUVBL1-RUVBL2-RAD51 DNA repair complex, thereby suppressing host DNA repair mechanisms. Therefore, drug design targeting LAM and LprG also needs to face challenges from other mechanisms of action and targeted drugs (219).

### 5.2 The relationship between LAM and LprG and vaccine preparation

An ideal tuberculosis vaccine would possess strong protective efficacy, stable and durable immunogenicity, and no adverse reactions. It could prevent tuberculosis through single or multiple immunizations. However, no vaccine with better protection or sustainability than Bacillus Calmette-Guérin (BCG) has emerged in clinical practice yet. Although some new candidate vaccines (e.g. Dendritic cell-targeted vaccines activate naive T cells to release cytokines and generate protective immunity) have shown specific effects in animal models, long-term experiments, follow-ups, and verifications are still needed to determine how to apply them to humans and whether they can replace BCG (215).

As one of the virulence factors of Mtb, LAM has the potential to become a vaccine. However, there is currently no consensus on how to reduce the virulence of LAM and how much attenuated LAM should be included. Moreover, the limited clinical trial data makes it difficult to support the effectiveness of this antigen against tuberculosis. Secondly, there are multiple issues in the development of LAM vaccines, including the lack of reliable preclinical evaluation indicators, suitable animal models for vaccine assessment, methods for simulating exposure, unified clinical trial endpoint standards, consistent evaluation environments, and complex immune populations (200).

Previous studies have indicated that due to the conservation of LprG, this protein is not easily degraded or subjected to structural and functional mutations. This makes it a potential key component for Mycobacterium vaccines, a hypothesis that has been further validated in subsequent experiments (201, 202). Moreover, even when used as an adjuvant rather than the primary immunogenic antigen in vaccines, LprG-based vaccines can remain relatively stable *in vivo* and effectively protect against Mycobacterium tuberculosis infection (203). These findings demonstrate that LprG, as a biomolecule, holds certain value in the study of immunotherapy for emerging renal tuberculosis.

In addition to LprG, LAM has unique biological characteristics that make it a potential new target for vaccine development. Studies have shown that antibodies against LAM can modulate the immune response to Mtb (204). Yan et al. constructed fusion proteins such as 38KD-MPT32-MPT64, CFP10-MTB81-EspC, and Ag85B-HBHA to improve the therapeutic effect by enhancing the antibody recognition ability of tuberculosis patients (205). However, as this study did not delve deeply into renal tuberculosis or extrapulmonary tuberculosis, further exploration is needed to construct fusion proteins and incorporate LAM to improve the diagnostic efficiency of renal tuberculosis. LAM can also serve as an adjuvant for BCG or as an immunogenicity modulator, becoming one of the intervention measures for tuberculosis treatment (206). Meanwhile, the technology of using LAM to prepare antibodies *in vitro* may become a new immunotherapy method for renal tuberculosis in the future (171).

At present, various new methods and ideas for vaccine research have been established. We believe that LAM will be utilized in the future for vaccine preparation research, enabling the conduct of clinical trials and marking a unique and exciting stage in tuberculosis vaccine research.

## 6 Summary

Renal tuberculosis, the second most significant infectious disease in the world with a high mortality rate, has the same characteristics as pulmonary tuberculosis but also has its characteristics in the field of urinary systems. Due to Mycobacterium tuberculosis's ability to effectively inhibit processes such as phagosome-lysosome fusion, phagosome acidification, and pro-inflammatory cytokine release, it prevents host cell death. Meanwhile, studies have shown that M. tuberculosis possesses certain mechanisms that can even acquire iron from host cells as a mode of host defense mechanism. Therefore, exploring new diagnostic and treatment approaches for tuberculosis is actually an exploration of the biological functional mechanisms of Mycobacterium tuberculosis (214). The exploration of new diagnoses and treatment ideas for pulmonary tuberculosis is, in fact, an extension of the exploration of diagnosis and treatment plans for renal tuberculosis and even urinary system tuberculosis. Extracellular vesicles can be found in various human body fluids and represent a type of cell secretion with high research potential. The research conclusions on its diagnosis and treatment of renal tuberculosis have more clinical guidance and practical effects.

In the research on the diagnosis of renal tuberculosis using LAM and LprG, these two biomarkers have their unique research value. LAM, as a characteristic virulence factor of Mycobacterium tuberculosis, can determine the presence and degree of infection. LprG, as the transport and riveting protein of LAM, can promote the function of LAM, but at the same time, it can also target and locate LAM through LprG. Due to the close relationship between LprG and LAM, utilizing LprG to identify and capture LAM has become a feasible diagnostic scheme design idea, thereby improving the detection rate.

Because LAM and LprG are biomarkers secreted by Mycobacterium tuberculosis (Mtb) and immune cells infected by Mtb, including macrophages, in extracellular vesicles. Therefore, the detection method using these two biomarkers effectively addresses the challenges encountered in traditional Xpert testing, including (1) The ability to detect only sputum samples; (2) Low sensitivity; (3) Long detection time, etc. At the same time, since exosomes can be found in various parts of the human body fluid, for patients with renal tuberculosis or other urinary system tuberculosis, and for patients with related immune deficiencies, tuberculosis detection through LAM and LprG is a suitable detection method.

In the therapeutic field, both of them serve as signature antigens of Mtb, showing promising prospects and research value in the development of vaccines, adjuvants, immunotherapy, and other treatment modalities. Moreover, relevant experimental results have been successfully implemented, yielding excellent outcomes.

The emergence of renal tuberculosis caused by drug-resistant Mycobacterium tuberculosis has further promoted research on extracellular vesicles and their contents in the diagnosis and treatment of renal tuberculosis. Although relevant experiments have been conducted on the use of extracellular vesicles as disease drug carriers and targeted therapies (207), targeted therapies for renal tuberculosis may be developed in the future. However, no related drugs have entered clinical trials, nor have their efficacy and economic benefits been widely accepted and recognized. Renal tuberculosis caused by multi-drug resistant Mycobacterium tuberculosis requires earlier diagnosis, treatment, and monitoring compared to ordinary renal tuberculosis to prevent severe sequelae and other adverse events (208, 209). The detection methods for LAM and LprG can utilize urine samples, which are easily obtained from patients with renal tuberculosis, thereby providing inherent advantages in early screening for renal tuberculosis or sputum-negative tuberculosis. Moreover, even for patients undergoing renal auto-nephrectomy, the detection of LAM and LprG can simplify disease detection due to their independence from sample timing and spatial constraints. The experiments summarized in this article involving children and HIV-infected tuberculosis patients have proven the above viewpoints. Meanwhile, some of the study populations selected also appear to suggest that the ability of LAM and LprG to accept non-sputum specimens is valuable in diagnosing renal tuberculosis.

At present, LprG is more commonly used as a means of localizing or capturing LAM in the detection of tuberculosis. The diagnostic capability of LprG alone for tuberculosis requires more experiments and research data for further evaluation. Moreover, the experimental designs of some researchers listed in this article have certain shortcomings, such as regional limitations or small sample sizes. Follow-up studies with multi-center, large sample sizes, and strict experimental operations are needed to confirm further the diagnostic and therapeutic value of LAM and LprG in pulmonary or extrapulmonary tuberculosis, including renal tuberculosis.

In the field of vaccine development for renal tuberculosis, both LAM and LprG have demonstrated sound biological and immunological effects *in vivo*, whether used as chimeric systems combined with other substances to prepare vaccines or as adjuvants for different vaccines. Moreover, even passive immunization with *in vitro*-prepared LAM antibodies can enable organisms to develop resistance against tuberculosis. We speculate that this may be related to the high conservation of LprG and its specific virulence factor role in Mycobacterium tuberculosis. These experiments suggest the possibility of conducting clinical research on LAM and LprG as new tuberculosis vaccines or immunotherapeutic agents in the future.

Although the two biomarkers present in EVs listed in this article are of great clinical value, we must also be aware of their current limitations in clinical practical application. These limitations include difficulties in isolation and purification, heterogeneity and specificity that may vary significantly between individuals, limitations in detection techniques, a lack of clinical trials and standardization, and the fact that LAM and LprG in EVs may fluctuate with disease progression. These issues may require further research to improve isolation and purification techniques, enhance detection sensitivity and specificity, standardize operating procedures, strengthen clinical validation, and integrate multi-omics analysis to develop high-throughput technologies to improve the detection rates of LAM and LprG. Meanwhile, due to significant differences between the results of LAM and LprG-related tests and traditional detection methods, healthcare workers in clinical settings have refused to diagnose tuberculosis using LAM and LprG, thereby withholding early anti-tuberculosis treatment for patients with positive LAM test results (217).

In summary, LAM and LprG in renal tuberculosis extracellular vesicles not only have the potential to serve as diagnostic markers but also as relevant therapeutic targets for drugs. They also have the potential to be used as vaccine formulations or adjuvants in the prevention of renal tuberculosis. Future research should further explore the clinical application value of these approaches to provide new strategies for the early diagnosis and precise treatment of renal tuberculosis.
